# Supplementary material for: Mitral Transcatheter Edge-to-Edge Repair and Clinical Value of Novel Echocardiographic Biomarkers: A Hypothesis-Generating Study
Source: Biomedicines. 2024 Aug 1;12(8):1710. doi: 10.3390/biomedicines12081710 (PMC11351940; doi:10.3390/biomedicines12081710)
Supplement: Supplementary file 1 [file biomedicines-12-01710-s001.zip › biomedicines-3079985-supplementary.pdf]

	Our cohort: all patients (N = 78)	EVEREST: organic and functional MR [20] (N = 78)	COAPT: functional MR [21] (N = 302)
Diabetes	43.5%	41%	35.1%
Ischemic heart disease	46.1%	84.2%	60.9%
Atrial fibrillation	62.8%	61.6%	57.3%
Chronic obstructive pulmonary disease	15.3%	34.6%	23.5%
Chronic Kidney disease	42.3%	23.1%	71.6%
NYHA 3 or 4	65.4%	89.8%	57.0%
Prior heart failure hospitalization	69.2%	65.0%	58.3%
EROA (cm^2)	0.37 (0.30-0.40)	-	0.41±0.15
LVEF (%)	50.0 [36.0;60.0]	54.4 ± 13.7	31.3 ± 9.1
LVEDV (ml)	120 [90.8;151]	166 ± 51	194.4±69.2
LVESV (ml)	61.0 [38.0;91.0]	80 ± 43	135.5 ± 56.1
LVEDD (mm)	54.9±8.87	-	62 ± 7
LVESV (mm)	38.0 [31.0;49.0]	39 ± 11	53 ± 9
PASP (mmHg)	48.5 [38.8;66.0]	-	44.0±13.4

Table S1. Baseline characteristics of our cohort, EVEREST and COAPT patients. MR, Mitral Regurgitation; NYHA, New York heart association; EROA, effective regurgitant orifice area; LVEF, left ventricular ejection fraction; LVED, left ventricular end-diastolic volume; LVESV, left ventricular end-systolic volume; LVEDD, left ventricular end-diastolic diameter; LVESD, left ventricular end-systolic diameter; PASP, pulmonary artery systolic pressure.
